# Feasibility and efficacy of helical tomotherapy in cirrhotic patients with unresectable hepatocellular carcinoma

**DOI:** 10.1186/s12957-015-0611-9

**Published:** 2015-06-15

**Authors:** Chun-Ming Huang, Ming-Yii Huang, Jen-Yang Tang, Shinn-Cherng Chen, Liang-Yen Wang, Zu-Yau Lin, Chih-Jen Huang

**Affiliations:** Department of Radiation Oncology, Kaohsiung Medical University Hospital, 100 Tzyou 1st Road, Kaohsiung, Taiwan; Department of Radiation Oncology, Faculty of Medicine, College of Medicine, Kaohsiung Medical University, Kaohsiung, Taiwan; Division of Hepatobiliary Medicine, Department of Internal Medicine, Kaohsiung Medical University Hospital, Kaohsiung, Taiwan; Department of Internal Medicine, Faculty of Medicine, College of Medicine, Kaohsiung Medical University, Kaohsiung, Taiwan; Cancer Center, Kaohsiung Medical University Hospital, Kaohsiung, Taiwan

**Keywords:** Helical tomotherapy, Hepatocellular carcinoma, Survival, Cirrhosis

## Abstract

**Background:**

This study is to evaluate the toxicity and outcomes of helical tomotherapy (HT) in patients treated for unresectable hepatocellular carcinoma (HCC).

**Methods:**

From March 2008 to September 2010, 38 patients with unresectable HCC were treated with HT. The median patient age was 67 years (range, 45–85). The median follow-up period was 17.2 months (range, 7–46). All patients had liver cirrhosis. Median radiation dose was 54 Gy (range, 46–71.8) delivered in 1.8 to 2.4-Gy fractions. The planning target volumes were 241.2 ± 153.1 cm^3^ (range, 45.8–722.4). Treatment responses were assessed in 3–6 months after HT.

**Results:**

There was a complete response in 2 patients (5.2 %), partial response in 18 patients (47.4 %), stable disease in 13 patients (34.2 %), and progressive disease in 5 patients (13.2 %). The median overall survival was 12.6 months, and 1- and 2-year overall survival rates were 56.2 and 31.7 %, respectively. Eastern Cooperative Oncology Group (ECOG score, *p* = 0.008), Child-Pugh classification (*p* = 0.012), albumin (*p* = 0.046), and hemoglobin (*p* = 0.028) were significant parameters that predicted primary tumor response to radiotherapy in multivariate analysis. ECOG score (*p* = 0.012), Child-Pugh class (*p* = 0.026), and response to radiotherapy (*p* = 0.016) were independent prognostic factors for overall survival in multivariate analysis. Responders had better overall survival than non-responders (23.6 vs. 5.8 months, *p* < 0.001). The 1- and 2-year overall survival rates for responders were 68.3 and 57 %, respectively, while for non-responders, they were 0 %. The 1- and 2-year local control rates were 88.2 and 82.3 %, respectively. Five patients (13.2 %) had grade 3 or greater liver toxicity, and one patient (2.6 %) had a grade 3 gastric ulcer. No treatment-related liver failure or death was documented in this study.

**Conclusions:**

Radiotherapy using HT seems to be a safe and effective treatment option for unresectable HCC patients. This study indicates that HT is a feasible treatment even in patients without good performance status and hepatic function reservation.

## Background

Hepatocellular carcinoma (HCC) is the fifth most frequently diagnosed cancer in men and the second reason of cancer-related mortality in the world [1]. In women, it is the seventh most common cancer and sixth leading cause of cancer death. Because of the prevalence of hepatitis B and C, the incidence of HCC is particularly high in East Asia and Africa [2]. Complete surgical resection and hepatic transplantation is considered a curative therapy for HCC, but only less than 15 % of patients with HCC are indicated for curative surgery because of tumor extent or compromised hepatic function [3, 4]. It remains challenging to treat patients with unresectable HCC, and most of them have dismal prognosis. Several other treatment modalities for patients with unresectable HCC, including percutaneous ethanol injection (PEI), radiofrequency ablation (RFA), and transcatheter arterial chemoembolization (TACE), seem to be more effective in smaller tumors and are contraindicated in patients with portal vein tumor thrombosis (PVTT), ascites, or biliary obstruction.

Given the risks of radiation-induced liver disease (RILD) and even liver failure and low tolerance of whole liver irradiation, radiotherapy (RT) has played a limited role in managing advanced HCC with conventional RT techniques [5]. The limitation is more prominent in HCC patients with underlying liver cirrhosis, which is prone to decompensated liver disease. With the advent of intensity-modulated radiotherapy (IMRT) and image-guided radiotherapy (IGRT), higher tumoricidal dosage could be more safely and precisely delivered to liver tumor and might theoretically increase local tumor control [6, 7]. Emerging evidence has shown that partial liver irradiation with three-dimensional conformal radiation therapy (3D-CRT) resulted in low toxicity and long-term tumor control in patients with unresectable HCC [8, 9]. However, the treatment techniques, fractionation, and total doses of RT for patients with HCC have varied greatly within different studies.

Helical tomotherapy (HT) uses modulated treatment apertures (defined by dynamic multi-leaf collimator) and dose rate to improve target conformality, dose homogeneity, and normal tissue sparing compared to 3D-CRT and/or IMRT. The on-board megavoltage computed tomography (CT) detection allows for daily setup verification, which can minimize planning target margins and irradiated normal tissues. With integrating IGRT with IMRT, HT could deliver higher dose to the relatively large tumor without increasing the normal tissue damage. However, there have been a few clinical reports regarding oncologic outcomes and tolerance of HCC patients receiving HT. This retrospective study aimed at evaluating the efficacy and toxicity of HT for cirrhotic patients with unresectable HCC.

## Methods

### Patient selection

The diagnosis of HCC was made according to histopathology (*n* = 13) or the typical radiologic features in a cirrhotic liver with or without an elevated serum alpha-fetoprotein (AFP) level (*n* = 25). Typical images to diagnose the presence of HCC in a mass lesion greater than 2 cm in greatest dimension with hypervascularity were taken. Unresectable tumor was defined by extensive tumor, major vascular invasion, or a poor medical condition that disallows surgical resection. The inclusion criteria in this study were primary HCC confirmed by biopsy or imaging, patients considered unresectable, and age >18 years. Patients who had extrahepatic metastases, Eastern Cooperative Oncology Group (ECOG) score ≧3, Child-Pugh class C disease or double cancer were excluded. Sixty-four HCC patients treated with HT were enrolled in our hospital between March 2008 and February 2011. Among them, 26 patients were excluded; 17 patients had evidence of distant metastases, 5 did not complete the planned RT, 2 had prior abdominal irradiation, and 2 had other malignancies. Finally, 38 patients were evaluated in the current study. Written informed consent was obtained from all the patients, and this retrospective study was approved by the Institutional Review Board of Kaohsiung Medical University Chung-Ho Memorial Hospital (KMUH-IRB-20120155).

### Treatment

CT simulation was performed for each patient with 5-mm slice thickness without contrast medium. All patients were immobilized with a posterior vacuum-lock body fixation device and an anterior vacuum-sealed cover sheet to reduce respiration movement. To further diminish liver motion by breathing, all patients were educated to breathe shallowly and not to fight with the compression of anterior cover sheet. The gross tumor volume (GTV), including PVTT, was delineated according to contrast-enhancing tumor exhibiting on diagnostic CT or magnetic resonance imaging (MRI) images. The whole intrahepatic tumor was included in the GTV with physician’s concern about tumor size, preserved liver function, and the irradiated volume of the liver. A margin of 1 to 1.5 cm was added to the GTV to cover microscopic tumor extension. Another 0.5 to 1 cm radical margin and 1 to 1.5 cm craniocaudal margin were added to count for organ motion and setup errors (planning target volume (PTV)). The entire liver was contoured, and the normal liver volume was defined as the whole liver volume minus the GTV. Treatment planning was generated using Tomotherapy planning software, version 4.0 (Tomotherapy, Madison, WI). Total prescribed dose depended on the individual physician’s decision [10]. HT was delivered once per day, five times a week. Before each treatment, a megavoltage CT scan was performed to correct the displacement of tumors and internal organs automatically or manually.

Prescribing tumor dose to the 95 % isodose line to encompass the PTV was required. The organs at risk used in current study were (1) spinal cord: maximum dose ≤45 Gy; (2) kidneys: mean dose to bilateral kidneys must be <15 Gy, and no more than 30 % of the volume of kidney can receive ≥20 Gy; (3) liver: mean normal liver dose must be ≤28 Gy, and no more than 30 % of normal liver can receive ≥30 Gy (V30 < 30 %); and (4) stomach and small bowels: maximum dose ≤45 Gy.

### Follow-up and response evaluation

The tumor response was evaluated by contrast-enhanced CT scans or MRIs at 3 to 6 months after the completion of RT. The response evaluation to criteria in solid tumors (RECIST) was used to determine the tumor response after irradiation [11]. A complete response (CR) represented a complete disappearance of the irradiated tumor. Partial response (PR) represented at least a 30 % reduction of tumor in the greatest dimension. Progressive disease (PD) was defined as at least a 20 % increase of tumor in the greatest dimension, and stable disease (SD) was defined as any tumor volume change other than PR or PD. Patients with CR or PR after RT were defined as responders, and non-responders represented patients with SD or PD. Local control was defined as image-based absence of disease progression. Patients with CR, PR, or SD were categorized as a condition of local control of disease.

Treatment associated side effects were evaluated weekly during RT and at each follow-up visit after treatment. Late toxicity was determined when toxicity occurred 3 months after the completion of treatment. The toxicity was assessed according to Radiation Therapy Oncology Group toxicity criteria [12]. Radiation-induced liver disease was defined as the development of nonmalignant ascites without disease progression and an anicteric increase in the alkaline-phosphatase level at least twofold or in the transaminase level at least fivefold after RT [13].

### Statistical analysis

Chi-square test, Fisher’s exact test, or *t* test was used to determine the association between tumor response and various parameters. Parameters with a *p* value less than 0.05 in univariate analysis were further evaluated in multivariate analysis, using logistic regression model. The overall survival (OS) was calculated from the start of RT to the date of death or last follow-up visit. Time to local failure was defined from the beginning of the treatment until progression. Rates were estimated using the Kaplan-Meier method and compared the effect of each variable on the survival using the log-rank test. Parameters with a *p* value less than 0.05 in univariate analysis were further evaluated in multivariate analysis, using Cox regression hazard model. Data analyses were conducted with the JMP software (version 9.0, SAS Institute Inc., Cary, NC). Results were considered significant at *p* values < 0.05.

## Results

### Patient characteristics

Thirty-one men and 7 women were enrolled. The median patient age was 67 years (range, 45–85). All patients had liver cirrhosis. 27 patients (71.1 %) were Child-Pugh class A, and 11 patients (28.9 %) were class B. There were 24 patients (63.2 %) with portal vein tumor thrombosis. Twenty-nine patients underwent median 3 cycles (range, 1–9) of TACE (range, 1–9), and 18 patients received PEI or RFA, but all of them experienced poor treatment response or disease progression. HT was delivered as the first treatment for 3 patients because of contraindications for other treatment modalities. The characteristics of the 38 patients are shown in Table 1.

**Table 1:** Baseline characteristics of patients

Variables	No. of patients (%)
Age (years)	
Median	67
Range	45–85
Gender	
Male	31 (81.6)
Female	7 (18.4)
ECOG score	
0–1	24 (63.2)
2	14 (36.8)
Etiology	
Hepatitis B	21 (55.3)
Hepatitis C	11 (28.9)
Alcohol	6 (15.8)
Child-Pugh classification	
A	27 (71.1)
B	11 (28.9)
Alpha-fetoprotein (ng/ml)	
Median	50.6
≤ 400/>400	23 (60.5)/15 (39.5)
Albumin (g/dL)	
Median	3.6
Range	2.39–4.54
Hemoglobin (g/dL)	
Median	11.4
Range	8.2–16
AJCC tumor stage	
T1–2	8 (21.1)
T3–4	30 (78.9)
Tumor size(cm)	
Median (range)	4.6 (2.5–16.7)
≤ 4/>4	10 (26.3)/28 (73.7)
PVTT	
Yes	24 (63.2)
No	14 (36.8)
Previous treatment	
TACE	29
PEI or RFA	18
None	3

*PVTT* portal vein tumor thrombus, *ECOG* Eastern Cooperative Oncology Group, *TACE* transcatheter hepatic arterial chemoembolization, *PEI* percutaneous ethanol injection, *RFA* radiofrequency ablation, *AJCC* American Joint Committee on Cancer

### RT parameters

The median RT dose was 54 Gy (range, 46–71.8 Gy). Because of different doses per fraction used in the study, the biologically effective doses (BED) were also assessed and are summarized in Table 2. The mean volume of PTV was 241.2 cm^3^ (range, 44.8–722.4), and the mean normal liver volume was 987.7 cm^3^ (range, 391.5–1515.4). The mean normal liver V30 was 12.8 % (range, 3–27 %). The mean dose to normal liver was 17.6 Gy (range, 6–28), and the average V10, V18, and V40 of normal liver were 64.6, 32.7, and 6.4 %, respectively. The mean doses to the right kidney, left kidney, small bowel, and stomach were 8.6, 3.2, 4.6, and 6.7 Gy, respectively.

**Table 2:** Summary of tomotherapy

Variables	
Total dose (Gy)	
Median	54
Range	46–71.8
BED10 (Gy_10_)	
Median	65.55
Range	40.35–85.72
RT duration (days)	
Median	37
Range	33–73

*BED*_*10*_ biologic effective dose, *RT* radiation therapy

### Tumor response rates

Median follow-up time was 17.2 months (range, 7–46). All patients were evaluable for the tumor response. There was CR in 2 patients (5.2 %), PR in 18 patients (47.4 %), SD in 13 patients (34.2 %), and PD in 5 patients (13.2 %). The objective tumor response rate was 52.6 %. Univariate analysis showed that ECOG score, Child-Pugh classification, albumin, hemoglobin, mean BED, and mean RT duration were statistically significant predictors of tumor response. ECOG score (*p* = 0.008), Child-Pugh classification (*p* = 0.012), albumin (*p* = 0.046), and hemoglobin (*p* = 0.028) remained significant in multivariate analysis.

The 1- and 2-year local control rates were 88.2 and 82.3 %, respectively. Patients with Child-Pugh B, AFP > 400 ng/ml, albumin ≤ 3.6 g/dL, hemoglobin ≤ 11 g/dL, or BED ≤ 65.5 Gy_10_ were more likely to relapse. In multivariate analysis, a higher BED remained significantly associated with a lower risk of relapse (hazard ratio (HR) = 0.78; 95 % confidence interval (CI), 0.69–0.92; *p* = 0.023). AFP (HR = 0.81; 95 % CI, 0.74–0.93; *p* = 0.011) and Child-Pugh classification (HR = 0.85; 95 % CI, 0.69–0.98; *p* = 0.022) were also associated with higher risk of local failure. Results are presented in Table 3.

**Table 3:** Predictive factors for local control

	Univariate analysis		Multivariate analysis	
Parameters	Hazard ratio (95 % CI)	*P* value	Hazard ratio (95 % CI)	*P* value
Age (≤67/>67) years	1.13 (0.49–2.78)	0.772	1.01 (0.97–1.07)	0.762
Gender (male/female)	0.75 (0.31–1.76)	0.519	1.33 (0.46–3.95)	0.585
ECOG score (0–1/2)	1.13 (0.48–2.84)	0.778	1.73 (0.66–4.94)	0.264
Etiology (viral/non-viral)	1.01 (0.79–1.27)	0.959	2.17 (0.61–8.28)	0.227
Child-Pugh class (A/B)	0.87 (0.74–0.96)	0.003	0.85 (0.69–0.98)	0.022
AFP (≤400/>400) ng/ml	0.83 (0.76–0.93)	<0.001	0.81 (0.74–0.93)	0.011
Albumin (>3.6/≤3.6) g/dL	0.88 (0.71–0.91)	0.049	1.26 (0.52–6.41)	0.231
Hemoglobin (>11/≤11) g/dL	0.72 (0.64–0.88)	0.044	1.41 (0.59–6.86)	0.213
PVTT (no/yes)	2.36 (0.87–6.39)	0.091	1.66 (0.68–5.12)	0.121
BED (>65.5/≤65.5) Gy_10_	0.78 (0.69–0.92)	0.023	0.81 (0.71–0.93)	0.032
RT duration (≤37/>37) days	1.07 (0.92–6.34)	0.029	1.52 (0.41–5.24)	0.512

*PVTT* portal vein tumor thrombus, *HCC* hepatocellular carcinoma, *ECOG* Eastern Cooperative Oncology Group, *BED* biologically effective dose, *RT* radiation therapy, *AFP* Alpha-fetoprotein

### Survival analysis and predictors of survival

At the time of analysis, 31 patients had died and 7 patients remained alive. The median survival time was 12.6 months (range, 3.6–40.9), with 1- and 2-year survival rates of 56.2 and 31.7 %, respectively.

**Fig. 1:**
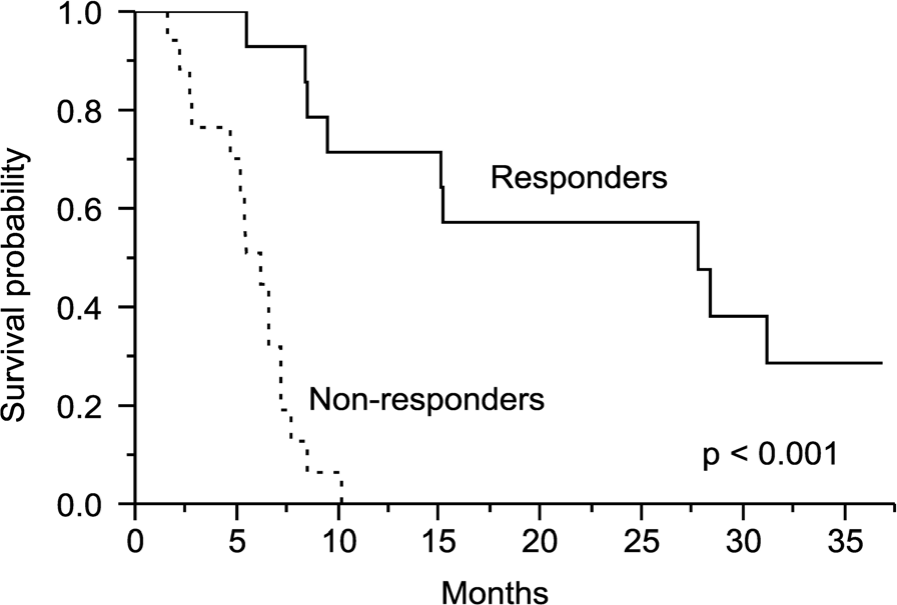
Overall survival rates according to the response of tumor. Responders had significantly better survival than non-responders (*p* < 0.001)

**Fig. 2:**
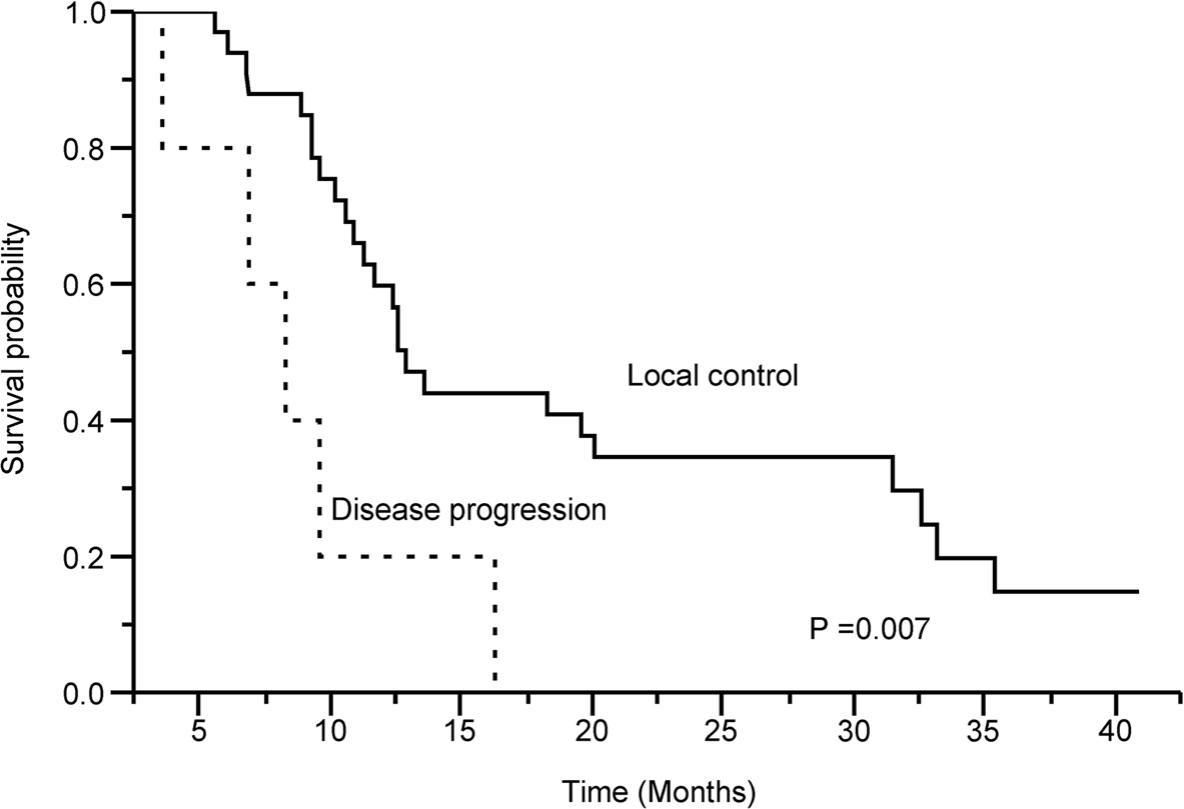
Patients remained local control after radiotherapy had significantly longer median survival time than patients with progressive disease (13.2 vs. 7.3 months, *p* = 0.007)

On the log-rank test, poor ECOG score, Child-Pugh class B, albumin ≤ 3.6 g/dL, and response to RT were significant unfavorable prognostic factors for overall survival. In Cox regression analysis, ECOG score (*p* = 0.012), Child-Pugh class (*p* = 0.026), local tumor control (*p* = 0.011), and response to radiotherapy (*p* = 0.016) were independent prognostic factors for overall survival (Table 4). For patients achieving tumor response (CR + PR), the median survival time was 27.8 ± 8.5 months, which was much higher than 6.2 ± 2.8 months of non-responders. The 1- and 2-year overall survival rates for responders were 68.3 and 57 %, respectively, while those without response were 0 % (Fig. 1). Patients remained local control after RT had significantly longer median survival time than patients with progressive disease (13.2 vs. 7.3 months, *p* = 0.007; Fig. 2).

**Table 4:** Predictive factors for overall survival

Factors	Median survival (months)	*P* value	
		Univariate analysis	Multivariate analysis
Age (years)		0.821	
≤ 67	8.5 ± 5.5		
> 67	7.2 ± 4.7		
Gender		0.225	
Male	8.5 ± 6.2		
Female	6.3 ± 2.8		
ECOG score		<0.001	0.012
0–1	9.5 ± 7.2		
2	3.8 ± 1.6		
Etiology		0.153	
Viral	8.5 ± 5.5		
Non-viral	6.6 ± 5.5		
Child-Pugh class		<0.001	0.026
A	8.5 ± 7.2		
B	5.2 ± 1.6		
AFP (ng/ml)		0.890	
≤ 400	8.5 ± 6.2		
> 400	7.2 ± 2.8		
Albumin (g/dL)		0.003	0.076
≤ 3.6	6.6 ± 2.8		
> 3.6	15.2 ± 6.2		
Hemoglobin (g/dL)		0.141	
≤ 11	6.6 ± 5.2		
> 11	9.5 ± 6.2		
PVTT		0.541	
Yes	10.2 ± 5.5		
No	6.6 ± 4.7		
BED (Gy_10_)		0.836	
≤ 65.5	7.2 ± 5.5		
> 65.5	15.1 ± 1.6		
RT duration (days)		0.443	
≤ 37	8.5 ± 5.5		
> 37	7.8 ± 2.8		
Tumor response		<0.001	0.016
Yes	27.8 ± 8.5		
No	6.2 ± 2.8		
Local control		0.007	0.011
Yes	13.2 ± 4.8		
No	7.3 ± 2.6		

*PVTT* portal vein tumor thrombus, *HCC* hepatocellular carcinoma, *ECOG* Eastern Cooperative Oncology Group, *BED* biologically effective dose, *SD* standard deviation, *AFP* Alpha-fetoprotein

### Treatment-related toxicity

The toxicities for all patients during RT and 2–3 months afterward are summarized in Table 5. Most of the acute gastrointestinal toxicities were self-limited. In terms of hepatic toxicities, five patients (13.2 %) experienced Common Terminology Criteria for Adverse Events (CTCAE) grade 3 or higher transaminase elevation and four of them simultaneously suffered from grade 3 or higher bilirubin elevation. One patient experienced RILD according to prior definition, and transaminase levels of the patient returned to normal under supportive care at 3 months after completion of RT. Normal liver volume for this patient was 423.6 cm^3^, and lesion size was 9.6 cm in diameter. RT parameters were as follows: prescription dose was 56 Gy in 25 fractions, mean dose to normal liver was 26.8 Gy, and V30 was 26.5 %. For gastrointestinal toxicities, only one patient experienced a gastric ulcer bleeding at 4 months after RT, but after proton pump inhibitor therapy, he recovered from the ulcer. Four patients had CTCAE grade 1 or 2 gastroduodenitis or ulcers, and all of them were transient. There was no treatment-related liver failure or death in this study.

**Table 5:** Adverse events in the study

	No. of patients with CTCAE grade (%)			
Adverse event	1 (%)	2 (%)	3 (%)	4 (%)
AST/ALT	21 (55.3)	6 (15.8)	4 (10.5)	1 (2.6)
Alkaline-phosphatase	3 (7.9)	–	–	–
Bilirubin	12 (31.6)	2 (5.3)	3 (7.9)	2 (5.3)
Gastroduodenitis	2 (5.3)	1 (2.6)	–	–
Gastric ulcer	2 (5.3)	–	1 (2.6)	–
Duodenal ulcer	1 (2.6)	1 (2.6)	–	–

*CTCAE* Common Terminology Criteria for Adverse Events, *AST* aspartate aminotransferase, *ALT* alanine aminotransferase

## Discussion

The indication of RT for patients with unresectable HCC has yet to be defined. HCC is a radiosensitive tumor, but it is within a considerably radiosensitive organ. In the past, RT has been considered as palliative and potentially toxic for HCC patients with underlying cirrhosis. Preservation of liver function and achievement of tumor control are equally important for their prognosis [14]. Combining intensity-modulated and image-guided radiotherapies, HT can deliver highly conformal radiation dose to liver tumors while sparing most of the normal liver parenchyma to reduce hepatic toxicity. In this study, low incidence of severe liver dysfunction was observed in HCC patients with underlying cirrhosis receiving HT and objective tumor regression after RT was associated with better prognosis.

For unresectable HCC patients receiving 3D-CRT, some studies reported tumor response rates ranged from 40 to 72.7 % [15–18]. Kong et al. [15] reported an objective response rate of 72.7 % to HT in 22 unresectable HCC patients. However, only 8 of 20 patients (36.4 %) had PVTT and may be biased to a high response rate. Kim et al. [16] reported that an objective response rate for primary liver tumors after 3D-CRT was 54.3 %, and for PVTT, a response rate was 39.0 %. Kang et al. [18] treated 27 HCC patients with IMRT and reported an objective response rate of 44.4 %. The study included 18 cases (66.6 %) with PVTT. Patients with HCC invading portal vein seem to have lower response rates to RT compared with values observed in patients with primary HCC. In our study, HT was delivered to 38 patients with unresectable HCC and 24 patients (63.2 %) had PVTT. We found a CR rate of 5.2 % (2/38), a PR rate of 47.4 % (18/38), and an objective response rate of 52.6 %. With relatively higher proportion of patients with PVTT, the response rate of our study is still encouraging.

The survival rate in our study is relatively low compared with values previously reported. Kong et al. [15] reported that 1- and 2-year overall survival rates were 86.4 and 69.1 %, for 22 HCC patients treated with tomotherapy. McIntosh et al. [19] performed IMRT using HT in 22 HCC patients with or without PVTT. The 1 year overall survival was 73 % for patients with Child-Pugh class A disease and 11 % for patients with Child-Pugh class B disease. Kim et al. [16] treated 70 HCC patients with or without PVTT by 3D-CRT and reported 1- and 2-year overall survival rates of 43.1 and 17.6 %, respectively. In our study, the 1- and 2-year overall survival rates were 56.2 and 31.7 %, respectively. The relatively low survival rates might be due to inclusion of a larger proportion of patients with poor prognostic factors such as PVTT, ECOG scale 2, and Child-Pugh class B disease. It might be those adverse factors that prompted physicians and patients to choose HT, aiming to reduce treatment toxicity with adequate tumor ablation dosage. Although this overall survival rate seems relatively low, it is not a low value when considering that most of the patient population in our study had advanced HCC and adverse factors.

Several studies have reported that primary tumor response was significantly associated with overall survival of patients with HCC following RT [15, 16, 18]. In our study, patients achieving objective tumor response (CR + PR) had a median survival of 27.8 ± 8.5 months, while non-responders had a median survival of 6.2 ± 2.8 months. The 1- and 2-year overall survival rate for responders was 68.3 and 57 %, respectively, while non-responders had dismal overall survival rates. This finding supports that RT can be one of the treatment options in unresectable HCC patients who failed to respond to previous treatments. It is also crucial to identify clinical or molecular parameters that can predict liver tumor response after RT.

A dose-response relationship in RT for HCC was shown in some studies. A higher radiation dose was associated with a higher response rate [20–22] and a higher survival rate [16, 17]. Some studies failed to show the correlation between radiation dose and survival [15, 23]. In our studies, we used BED to analyze the correlation between radiation dose, tumor response, and patient survival because of a variety of fraction sizes ranging from 1.8 to 2.4 Gy. The response rates in patients receiving ≤65.5 Gy_10_ and >65.5 Gy_10_ were 44.4 and 59.1 %, respectively (*p* = 0.547). However, the mean BED of 65.1 ± 18.5 Gy in primary tumor responders was significantly lower than that of 70.1 ± 16.8 Gy in non-responders (*p* = 0.045). The mean duration of RT in tumor responders was shorter than that in non-responders (36.8 vs. 42.4 days, *p* = 0.045). As a whole, a higher BED with a shorter period of RT seemed to result in higher tumor response rate. Patients receiving >65.5 Gy_10_ or completing RT ≤37 days had longer median survival than their respective counterparts. However, it might be due to the small sample size and heterogeneity in studied cases that these differences were not statistically significant.

In addition to BED, ECOG score 2, Child-Pugh class B, hypoalbuminemia, and anemia were factors associated with poor RT response in our study. Increasing evidence has shown that the presence of a systemic inflammatory response correlates with poor prognosis in patients with advanced cancer [24]. A simple inflammation-based scoring system, combining C-reactive protein and serum albumin, is associated with prognosis in patients undergoing cancer treatments [25, 26]. In our study, hypoalbuminemia was not only associated with non-response to RT but also with a low survival rate. Therefore, correction of status of hypoalbuminemia in HCC patients might be beneficial.

Anemia is common in cancer patients and is considered to induce tumor hypoxia, which can contribute to radio-resistance. In addition, numerous studies have shown that anemia is an independent adverse predictor for overall survival and local control at various tumor sites [27–29]. We found that patients with hemoglobin >11 g/dL had better primary tumor response rates following RT (61.1 vs. 23.1 %, *p* = 0.035). Median survival rate in patients with hemoglobin >11 g/dL was 9.5 months, which was longer than 6.6 months in patients with hemoglobin ≤ 11 g/dL. However, the difference failed to show statistical significance due to small sample size.

The potential acute toxicities of HCC patients undergoing RT are gastrointestinal complications such as appetite loss and gastroduodenal ulcer or bleeding and hepatic toxicity such as RILD, which was considered the most dismal toxicity due to high fatality. In our study, gastrointestinal toxicities we encountered were manageable. The incidence of RILD ranged from 0 to 15.4 % [8, 9, 15], and in our study, RILD was found in one patient (2.6 %), which was low compared with values previously reported. Several studies correlated various dose-volumetric parameters with the risk of RILD. These included the mean dose to the normal liver, percentage of the normal liver volume receiving ≥30 Gy, percentage of the total liver volume receiving ≥30 Gy, and the normal tissue complication probability estimates [30–32]. However, the RT technique used in these studies was mainly 3D-CRT. It might be inappropriate to apply these findings to patients receiving IMRT, which has smaller radiation dose in the liver compared with 3D-CRT [33]. There is a need of further investigations about the impact of IMRT on RILD.

3D-CRT and IMRT are able to deliver a conformal radiation dose to cover the target volume while sparing the critical organs appears attainable [7, 33]. Rotational IMRT modalities, such as HT, are modern image-guided intensity-modulated RT techniques. These complex rotational IMRT machines are able to deliver highly conformal dose distributions and can spare critical organs to a greater extent [14, 15, 19]. Several data have shown that HT has dosimetric advantages over IMRT based on comparisons in treatment planning. However, there have been few reports on clinical outcomes for HCC patients undergoing HT. We present our data and compare it with other published studies. Table 6 summarizes the treatment outcomes and toxicity of previously published studies using HT in the treatment of HCC patients.

**Table 6:** Comparison of the literatures for helical tomotherapy in patients with unresectable hepatocellular carcinoma

Reference	No.	Fraction size (Gy)	Total dose (Gy)	Target of RT	Response (%)	Median Survival (months)	RILD (%)	Grade III toxicity	Combined therapy
McIntosh [19]	20	2.5	50	PVTT(+) 40 %	PR 6.2	9.6	N/A	UGI bleeding 1/20	Capecitabine
				PVTT(−) 60 %	SD 87.5				
Chi [34]	23	2.5–4.5	52.5 (median)	N/A	CR 8.6	16	1/23	UGI bleeding 2/23	Sunitinib
					PR 65.4			Hematology 6/23	
Kim [14]	35	4.5–6	50 (range, 45–60)	PVTT(+) 100 %	CR 14.3	12.9	0/35	GI bleeding 2/35	Capecitabine
					PR 28.6	Responder 13.9		Hematology 3/35	
						Non-responder 6.9			
Kong [15]	20	1.8–4	50–57.5 (range, 30–60)	PVTT(+) 36.4 %	CR 18.2	14.4	1/20	None	None
				PVTT(−) 63.6 %	PR 54.5	Responder 87.8^a^			
						Non-responder 65.6^a^			
Current study	38	1.8–2.4	54 (range, 46–71.8)	PVTT(+) 63.2 %	CR 5.2	12.6	1/38	1/38	None
				PVTT(−) 36.5 %	PR 47.4	Responder 27.8			
						Non-responder 6.2			

*RT* radiation therapy, *PVTT* portal vein tumor thrombus, *N/A* no data, *CR* complete response, *PR* partial response, *SD* stable disease, *RILD* radiation-induced liver disease, *GI* gastrointestinal

^a^1-year overall survival rate (%)

Some limitations exist in the current study. First, because this was a retrospective study, heterogeneity of study population and dose fractionation schedules were present. The tumor response to RT might be influenced by fractionated RT dose, but no consensus has been reached on a standard radiation dose fractionation schedule for patients with HCC until now. Thus, further research on optimal fraction size and total dose should be conducted. Second, the sample size was small and this may minimize the detection of small but clinically important parameters. Third, the follow-up period was not long. Therefore, long-term toxicity and quality of life of patients receiving HT for HCC cannot be evaluated.

## Conclusions

Using HT to treat patients with unresectable HCC, we found that treatment response and overall survival rates were good with a manageable toxicity even in patients with poor performance status and PVTT. For responders, the median survival duration was 27.8 months with 1- and 2-year overall survival rates of 68.3 and 57 %, respectively. The promising results indicate that stricter patient selection will maximize potential benefits of unresectable HCC patients receiving RT. Further large-scale, prospective randomized studies are needed to address the efficiency and optimal dose fraction schedules of RT.

## Abbreviations

AFP: alpha-fetoprotein
BED: biologically effective dose
CT: computed tomography
CR: complete response
ECOG: Eastern Cooperative Oncology Group
GTV: gross tumor volume
HCC: hepatocellular carcinoma
HT: helical tomotherapy
IMRT: intensity-modulated radiotherapy
IGRT: image-guided radiotherapy
OS: overall survival
PEI: percutaneous ethanol injection
PVTT: portal vein tumor thrombosis
PR: partial response
PD: progressive disease
RT: radiotherapy
RILD: radiation-induced liver disease
RFA: radiofrequency ablation
SD: stable disease
TACE: transcatheter arterial chemoembolization
3D-CRT: three-dimensional conformal radiotherapy
